# RIPK3-MLKL-mediated necroinflammation contributes to AKI progression to CKD

**DOI:** 10.1038/s41419-018-0936-8

**Published:** 2018-08-29

**Authors:** Hui Chen, Yulu Fang, Jianfeng Wu, Hong Chen, Zhenhuan Zou, Xiaohong Zhang, Jing Shao, Yanfang Xu

**Affiliations:** 1 Department of Nephrology, First Affiliated Hospital, Fujian Medical University, Fuzhou, 350005 China; 2 School of Life Sciences, Xiamen University, Xiamen, 361005 China; 3 Department of Pathology, First Affiliated Hospital, Fujian Medical University, Fuzhou, 350005 China; 4 Institute for Immunology, Tsinghua University School of Medicine, Beijing, 100084 China

## Abstract

Necroptosis predominates functionally over apoptosis in the pathophysiology of renal ischemia-reperfusion injury (IRI). Inhibition of the core components of the necroptotic pathway—receptor-interacting protein kinase 1 (RIPK1), RIPK3 or mixed lineage kinase domain-like protein (MLKL) reduced renal injury after ischemia/reperfusion (IR). Necrosis can initiate inflammation, which enhances necrosis in a positive feedback loop, subsequently leading to triggering more inflammation, termed as necroinflammation. However, the mechanisms underlying necroinflammation driven by renal tubular cell necroptosis in progression of AKI to CKD are still largely unknown. Here we showed that the upregulated expression and interactions between RIPK3 and MLKL induced necroptosis of renal proximal tubular cells and contributed to NLRP3 inflammasome activation under the conditions of IRI. Gene deletion of *Ripk3* or *Mlkl* ameliorated renal tubular cell necroptosis, macrophage infiltration and NLRP3 inflammasome activation with a reduction in caspase-1 activation and maturation of IL-1β, and then finally reduced interstitial fibrogenesis in the long term after IRI. Bone marrow chimeras confirmed that RIPK3-MLKL-dependent necroptosis is responsible for the initiation of the early renal injury after IRI, and then necroptosis triggered NLRP3 inflammasome activation, which subsequently accelerates necroptosis and triggers more inflammation in an auto-amplification loop. These data indicate that necroinflammation driven by RIPK3-MLKL-dependent necroptosis plays a crucial role in the progression of IRI to CKD.

## Introduction

Acute kidney injury (AKI) confers a greater risk of progression to chronic kidney disease (CKD). However, since there exists a causal link between AKI and CKD, the underlying mechanism are not fully illustrated and an effective therapeutic interventions specifically targeting this progression is still lacking^1^. The infiltration with interstitial inflammatory cells are reported as a universal cause in failing kidneys of CKD patients, which suggests that sterile inflammation has a critical role in CKD progression^2,3^. Sterile inflammatory responses induced by cellular injury are mainly associated with necrosis, and the necrotized cells release danger-associated molecular patterns (DAMPs)^4,5^. Subsequently, DAMPs activate the innate immunity to produce more inflammatory cytokines, which in turn aggravate necrosis and triggers more inflammatory reactions^6,7^. This positive feedback loop of inflammation in AKI, termed as necroinflammation, may eventually lead to renal failure^2,4^. However, the molecular mechanisms that regulate necroinflammation in the progression of AKI to CKD remain unclear.

It has been well delineated that necroptosis is a type of regulated and lytic cell death involving specific necrosome formation, containing receptor-interacting protein kinase (RIPK) 1, RIPK3, and mixed lineage kinase domain-like protein (MLKL)^8,9^. Upon phosphorylation of MLKL by RIPK3, the oligomerized MLKL leads to plasma membrane rupture by directly disrupting the membrane bilayer^9,10^. Both the inhibition of RIPK1(Nec-1)^11^ and deletion of RIPK3 or MLKL were shown to alleviate the renal dysfunction after IRI, supporting the critical role of necroptosis in the pathological processes of AKI^12–15^. Our previous study showed that cisplatin nephrotoxicity is involved in necroptosis of tubular cells, triggering necroinflammation^16^. Deletion of caspase 8 leads to severe chronic inflammation, whereas double deletion of RIPK3 and caspase 8 prevents this inflammatory induction^17,18^. These studies suggested that necrosome components control the inflammatory reactions.

Necroptosis could also trigger innate immunity by releasing DAMPs into extracellular space via the ruptured plasma membrane^6,7^. NOD-like receptors (NLRs) are pattern-recognition receptors and could efficiently sense the intracellular signals such as DAMPs including the chromatin regulator high mobility group box 1 (HMGB1)^6,19,20^. NLRP3 is one among the well-studied members of NLRs. NLR-forming inflammasomes are activated through NLRP3 and recruit apoptosis-associated speck-like protein containing a caspase activation and recruitment domain (ASC). Following inflammasome activation, the recruitment of procaspase 1 induces auto-proteolytic conversion of the pro-enzyme into active caspase 1, which in turn leads to cleavage and subsequent release of interleukin-1β (IL-1β) and IL-18^20–22^. This has been considered as an important common pathway of the inflammation–fibrosis cycle in the progression of AKI to CKD^2^. Interestingly, RIPK3-MLKL signaling can also promote inflammation by activating NLRP3 inflammasome and triggering caspase-1 processing to secret mature IL-1β in macrophages^17,23,24^. The MLKL-oligomerization and further translocation to cellular membranes are not only essential for necroptosis^25,26^ but also for NLRP3 inflammasome activation^24^. Thus, signal transduction of necrosome components directly regulates necrosis and inflammation.

However, the role of RIPK3-MLKL-mediated necroptosis and NLRP3 inflammasome activation in progression of AKI to CKD remains unclear. Ischemia-reperfusion injury (IRI), regarded as a major cause of AKI, can occur during shock, sepsis, and renal transplantation. Here we used mice IRI models to examine the role of RIPK3-MLKL-mediated necroinflammation during CKD progression post-AKI.

## Results

### Deletion of *Ripk3* or *Mlkl* genes reduced deterioration of renal function after IRI

**Fig. 1:**
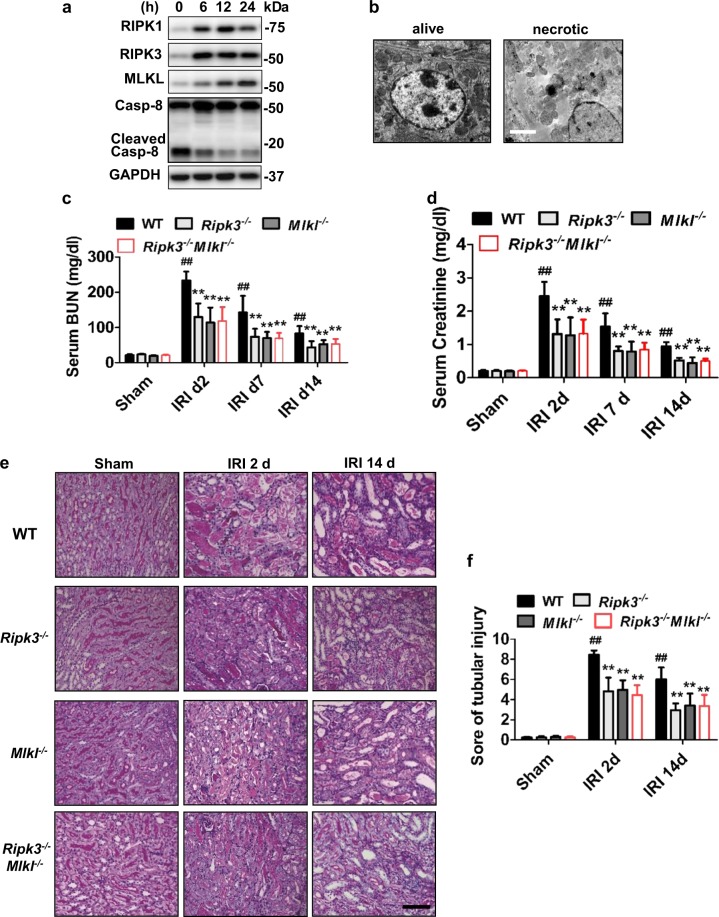
Poor outcomes of IRI were attenuated in *Ripk3* or/and *Mlkl* deficiency mice. **a**–**f** The kidneys and blood samples in mice were harvested at the indicated time following IRI (*n* = 8). **a** Freshly isolated proximal tubules were collected for Western blot to analyze of RIPK1, RIPK3, MLKL and caspase 8. **b** Representative electron micrographs of alive and necrotic PTCs are shown. Bar = 2 μM. **c**, **d** Serum BUN and creatinine levels were detected. **e** Representative images of PAS-stained kidney sections are shown. Bar = 100 μM. **f** Histologic damage in the outer medulla after IRI were scored by Periodic acid–Schiff (PAS) staining at 2 and 14 days after IRI. The degree of renal injury was assessed morphologically in the outer medulla regions of PAS-stained kidney sections. For counting, ten randomly selected fields per kidney were used. The percentage of tubules that displayed tubular necrosis, cast formation, and tubular dilation was scored semiquantitatively. ^##^*P* < 0.01 vs. Sham IRI, **P < 0.01 vs. WT IRI

We used mouse model with severe (40 min) period of bilateral renal pedicle clamping before reperfusion to examine the progression of AKI to CKD, which is consistent with some previously reported studies^12,13,27^. Inhibition of caspase-8 or induction of RIPK3 can initiate necroptosis^8,18^. The levels of three core components of necrotic pathways, RIPK1, RIPK3 and MLKL significantly increased during 6–24 h of ischemia developed after the beginning of reperfusion, while cleaved caspase-8 was reduced (Fig. 1a). The nuclear swelling and loss of cell organelles, which was necrotic but not apoptotic phenotype, were detected in the tissue sections (Fig. 1b). The serum concentrations of creatinine and BUN were significantly lower in *Ripk3* deficiency (*Ripk3*^−^/^-^), *Mlkl* deficiency (*Mlkl*^−/−^) and *Ripk3/Mlkl* double deficiency (*Ripk3*^−/−^*Mlkl*^−/−^) mice than all WT mice from day 2 to day 14 after IRI (Fig. 1c, d). Here we also found that at 14 days following IRI, BUN and Cr did not completely normalized to baseline (Fig. 1c, d). In line with this finding, as shown in representative photographs, WT mice exhibited severe tubular necrosis, cast formation, tubular dilation and interstitial inflammation at 2 and 14 days after IRI, which were attenuated in *Ripk3*^−/−^, *Mlkl*^−/−^, and *Ripk3*^−/−^ *Mlkl*^−/−^ mice (Fig. 1e). The cumulative scores of histologic damage assessed as described^13^ at 2 and 14 days post-IRI (Fig. 1f). Although RIPK3 may have other substrate^28^, however, *Ripk3*^−/−^ and *Ripk3*^−/−^*Mlkl*^−/−^ mice did not showed stronger renal protection after IRI compared with *Mlkl*^−/−^ mice (Fig. 1f). These results showed that inhibition of RIPK3-MLKL-mediated necroptosis reduced renal tubular damage and inhibited AKI progression.

### RIPK3 and MLKL contributed to the development of renal tubular interstitial fibrosis after IRI

**Fig. 2:**
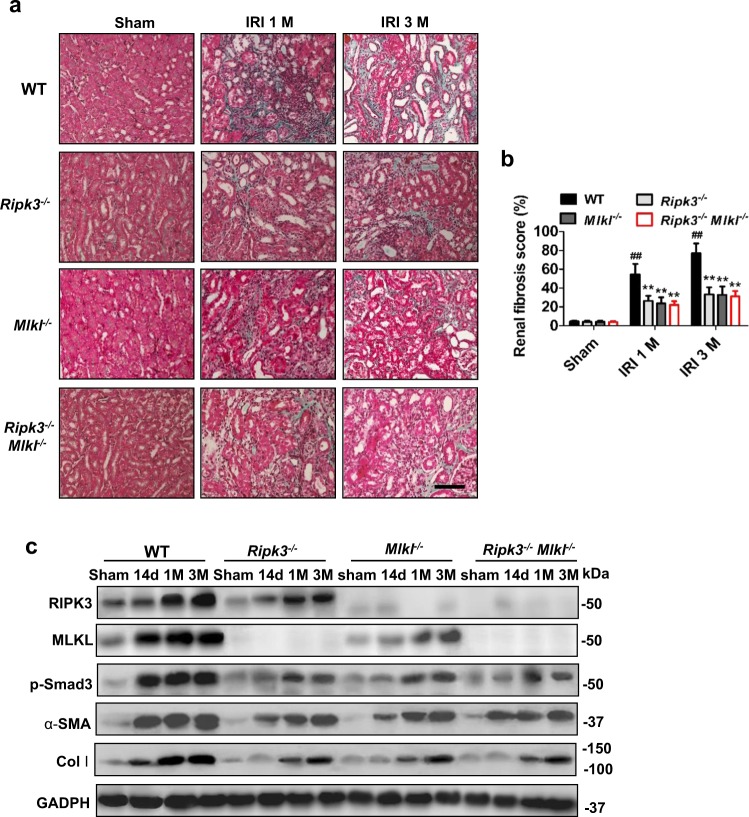
Deletion of *Ripk3* or/and *Mlkl* genes reduced renal fibrosis after IRI. All mice were subjected to IRI with 40-min ischemia and then were fed with normal rodent chow for the indicated time. **a** Kidney morphology by Masson’s trichrome staining. Representative micrographs in kidney sections. Bar = 100 μM. **b** Renal fibrosis scores obtained from Masson’s trichrome-stained sections were analyzed with Image J. *n* = 6. ^##^*P* < 0.01 vs. WT sham group; **P < 0.01 vs. WT IRI. **c** Representative immunoblot for RIPK3, MLKL, p-Smad3, α-SMA, Col I, and β-actin by western blot in total kidney lysates. *n* = 6

To explore the relationship between necroptosis and long-term renal outcome after AKI, we compared tubulointerstitial fibrosis with 40-min ischemia in WT, *Ripk3*^*−/−*^, *Mlkl*^−/−^ and *Ripk3*^−/−^*Mlkl*^−/^^−^ mice. The extent of renal fibrosis post IRI were evaluated by Masson’s trichrome staining and scored as described^27^. In this study, our results showed that 40-min renal ischemia led to maladaptive repair as shown by the scores of fibrosis. The progressive increase in interstitial fibrosis throughout 3 months after IRI were significantly ameliorated in *Ripk3*^−/−^, *Mlkl*^−/−^ and *Ripk3*^−/−^*Mlkl*^−/−^ mice (Fig. 2a, b). The protein levels of fibrotic markers, α-smooth muscle actin (α-SMA) and collagen I (Col I) were increased more significantly in WT mice in a time-dependent manner from 2 weeks to 3 months post IRI than those in *Ripk3*^−/−^, *Mlkl*^−/−^ and *Ripk3*^−/−^ *Mlkl*^−/−^ mice (Fig. 2c). The phosphorylation level of Smad3 (p-Smad3) was also increased in WT mice, but significantly reduced in *Ripk3*^−/−^, *Mlkl*^−/−^ and *Ripk3*^−/−^*Mlkl*^−/−^ mice (Fig. 2c).

To further investigate whether *Ripk3* and *Mlkl* deficiency could still reduce renal fibrosis progression after the similar initial renal injury, WT mice group were subjected to renal pedicle clamping for 35 min before reperfusion; whereas *Ripk3*^−/−^, *Mlkl*^−/−^ and *Ripk3*^−/−^*Mlkl*^−^^/−^ groups were subjected to 43-min renal ischemia. Parameters for assessing the loss of kidney function (elevated serum creatinine and urea) and histological changes in kidney at day 1 in these mice were comparable (data no shown). Remarkably, *Ripk3*^−/−^, *Mlkl*^−/−^ and *Ripk3*^−/−^*Mlkl*^−/−^ mice with 43-min ischemia displayed lower scores of fibrosis and lower levels of fibrotic markers than *WT* mice with 35-min ischemia (Supplementary Fig. S1a–c). Interestingly, there is no statistical significance in renal fibrosis among these *Ripk3*^−/−^, *Mlkl*^−/−^ and *Ripk3*^−/−^*Mlkl*^−/−^ mice with the same period of renal ischemia (Fig. 2a–c, Supplementary Fig. S1a–c). Collectively, these data indicated that both RIPK3 and MLKL contributed to progression of renal fibrosis post IRI.

### Necroptosis promoted macrophage recruitment post IRI

**Fig. 3:**
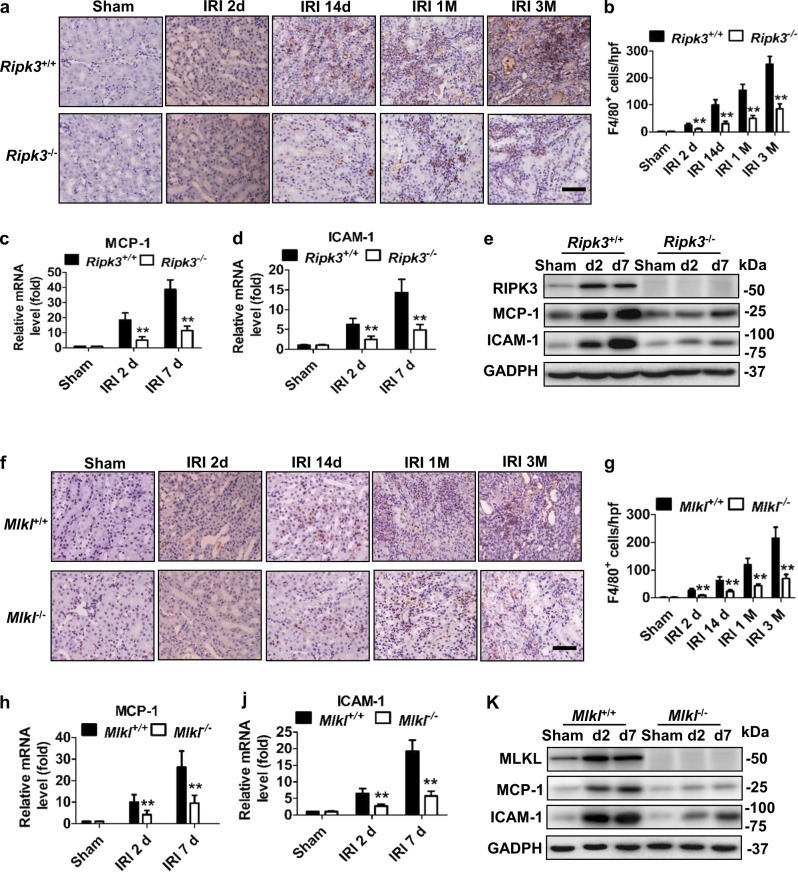
*Ripk3*^*−/−*^ and *Mlkl*^−/−^ mice displayed reduced tubulointerstitial inflammation post IRI. All mice were subjected to IRI with 40-min ischemia. **a**, **f** Representative images of immunohistochemistry of kidney tissues with the monocytes-macrophage marker F4/80^+^ at 2, 14 days and 1, 3 months following IRI. Bar = 100 μM. **b**, **g** The number of F4/80-positive cells per hpf was quantified. Quantitative RT-PCR (**c**, **d**, **h**–**j**) and western blot analysis for RIPK3, MLKL ICAM-1 and MCP-1 (**e**, **k**) of the freshly isolated renal tubules from mice on 2 days and 7 days after IRI. *n* = 6. **P < 0.01 vs. WT group

Inflammation plays a pivotal role in the pathophysiology of IRI and influences the severity and prognosis of AKI^29,30^. In order to assess the inflammation in tissue, we used F4/80 as a marker to indicate the monocytes/macrophages infiltration in injured kidneys. The number of F4/80^+^ cells mildly increased at early days post IRI, significantly increased at day 14 and persistently increased up to 3 months after IRI in WT mice. The infiltration of monocytes/macrophages was significantly reduced in *Ripk3*^−/−^ (Fig. 3a, b) and *Mlkl*^−/−^ mice (Fig. 3f, g) in comparison with WT mice. Interestingly, *Ripk3*^−/−^ and *Mlkl*^−/−^ mice with 43-min ischemia also displayed less monocytes/macrophages infiltration in kidney than WT mice with 35-min ischemia (Supplementary Fig. S2a–d).

Since monocyte chemotactic factor -1 (MCP-1) and intercellular adhesion molecule-1 (ICAM-1) play a role in mediating the migration of immune cells, we investigated ICAM-1 and MCP-1 expression in renal tubules after IRI. Similarly, significant increase of MCP-1 and ICAM-1 mRNA and protein in the freshly isolated renal proximal tubules from mice at 2 and 7 days after IRI were also found in all WT mice, whereas their expression were reduced in *Ripk3*^−/−^ (Fig. 3c–e) and *Mlkl*^−/−^ (Fig. 3h–k) mice. These data demonstrated that persistent inflammation in post severe IRI kidneys could result from the initiation of necroptosis that activated the migration and recruitment of inflammatory cells.

### *Ripk3* and *Mlkl* deficiency inhibited NLRP3 inflammasome activation post IRI

**Fig. 4:**
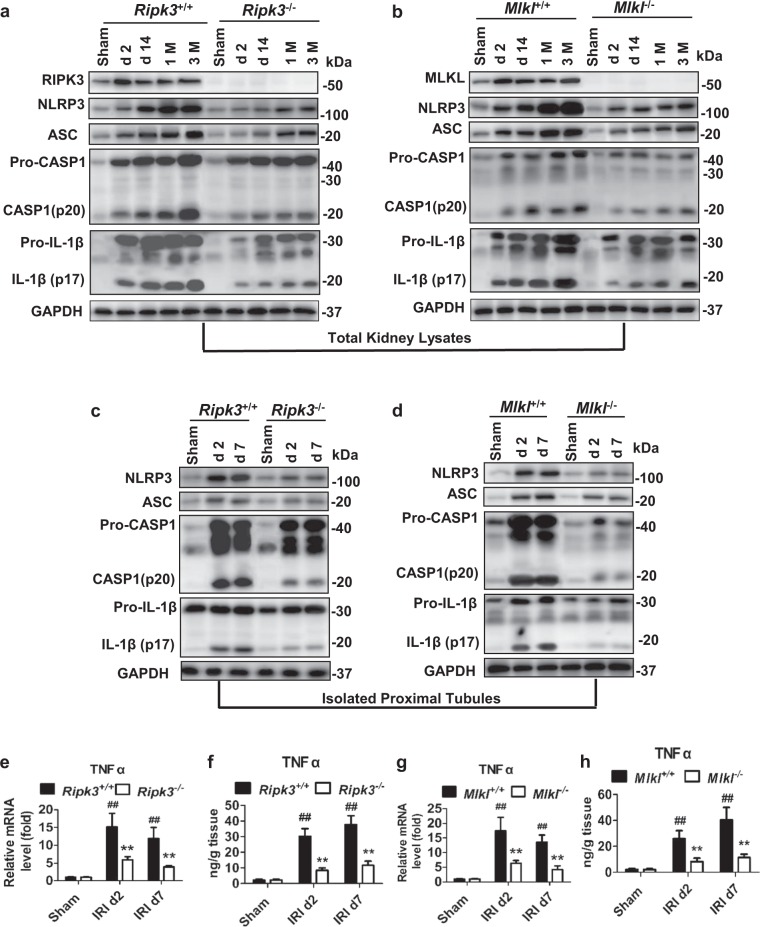
Deletion of *Ripk3* or *Mlkl* genes limited NLRP3 inflammasome activation post IRI. All mice were treated as Fig. 3. **a**, **b** Expression of NLRP3, ASC, the active caspase-1 and the mature (processed) IL-1β in total lysates of kidneys were assessed by Western blot analysis. GADPH was used as the loading control. *n* = 4. **c**, **d** Expression of NLRP3, ASC, the active caspase-1 and the mature (processed) IL-1β in renal proximal tubules were also assessed by western blot analysis. *n* = 4. **e**, **g** Quantitative RT-PCR analysis for TNFα of the freshly isolated renal tubules from mice on 2 days and 7 days after IRI. *n* = 4. **f**, **h** TNFα in total lysates of kidneys was detected by ELISA. *n* = 5. ^##^*P* < 0.01 vs. sham group; **P < 0.01 vs. WT group

The NLRP3 inflammasome activation and active mature forms of IL-1β and IL-18 sectetion contribute to the progression of AKI to CKD^1,3,4,30^. Upregulation of NLRP3 and ASC, caspase-1 activation and a subsequent increase of mature cleaved IL-1β were observed at day 2 and progressively increased over a 3-month time course after IRI in WT mice. In contrast, NLRP3 inflammasome activation was significantly reduced in *Ripk3*^−/−^ (Fig. 4a) and *Mlkl*^−/−^ mice (Fig. 4b) mouse littermates after the same peroid of renal ischemia with wt mice. Strikingly, *Ripk3*^−/−^ and *Mlkl*^−/−^ mice with 43-min ischemia also dramatically reduced renal NLRP3 inflammasome activation at 1 and 3 months compared to WT mice with 35-min ischemia (Supplementary Fig. S3a, b). Therefore, RIPK3 and MLKL not only contributed to necroptosis, but also to NLRP3 inflammsome activation during the progression from IRI to CKD.

Furthermore, we determined whether NLRP3 inflammasome activation existed in tubular cells after IRI. Since, necroptosis could be seen only in an early stage of IRI, we isolated proximal tubules from kidneys at 2 days and 7 days after IRI. Notably, NLRP3 inflammasome was also activated in proximal tubules, which could be attenuated by *Ripk3* or *Mlkl* deletion (Fig. 4c, d). Thus, RIPK3 and MLKL plays an important role in the activation of NLRP3-inflammasome in renal tubules and inflammatory cells post AKI. Since TNFα is the most important factor to trigger necroptosis^8–10^, we further showed necroptosis promoted TNFα mRNA and protein level in renal proximal tubules (Fig. 4e–h), which could enhance necroptosis in a positive feedback. These data indicated that RIPK3-MLKL-dependent necroptosis contributed to necroinflammation during IRI progression.

### Bone marrow-derived cells with *Ripk3* or *Mlkl* deficiency contribute to inhibiting IRI progression to CKD

**Fig. 5:**
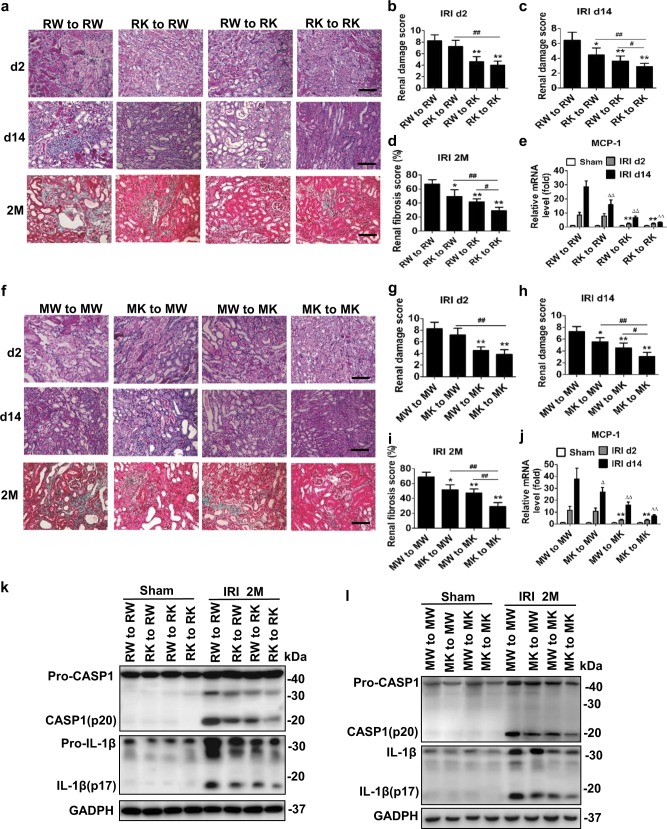
Bone marrow-derived cells with *Ripk3* or *Mlkl* deficiency reduced renal fibrosis and inflammasome activation in progression of IRI to CKD. Chimeric mice were created, in which the BM was replaced with donor BM cells from WT or from *Ripk3*^*−/*^^−^ or *Mlkl*^−/−^ mice. Irradiated *Ripk3*^+/+^ and *Ripk3*^*−/−*^ mice were reconstituted with either *Ripk3*^+/+^ or *Ripk3*^−/−^ BM cells, and similar experiments were performed in *Mlkl* background mice. **a** Representative kidney sections (Bar = 100 μM), and (**b**–**d**) quantification of renal injury in *Ripk3* BM chimeric mice at 2,14 days and 2 months after IRI. *n* = 6. *P *<* 0.05,**P < 0.01 *vs. RW* to *RW* chimeric mice; ^##^*P* < 0.01, vs. *RK* to *RW* chimeric mice; ^#^*P* < 0.05, vs. *RW* to *RK* chimeric mice. **f** Representative kidney sections (Bar = 100 μM) and (**g**–**i**) quantification of renal injury in *Mlkl* BM chimeric mice at 2,14 days and 2 months after IRI. *n* = 6. *P *<* 0.05, **P < 0.01 vs. *MW* to *MW* chimeric mice; ^##^*P* < 0.01, vs. *MK* to *MW* chimeric mice; ^#^*P* < 0.05, vs. *MW* to *MK* chimeric mice. **e**, **j** Quantitative RT-PCR analysis for MCP-1 of the total kidney lysates from mice on 2 and 14 days after IRI. **P < 0.01, vs. *RW* to *RW* or *MW* to *MW* chimeric mice on day 2 IRI; ^∆^*P* < 0.05, ^∆∆^*P* < 0.01, vs. *RW* to *RW* or *MW* to *MW* IRI chimeric mice on day 14 IRI. *n* = 6. **k**, **l** Representative Western blot image of active caspase-1 and mature (processed) IL-1β were shown. GADPH was used as the loading control. *n* = 4. RW *Ripk3*^+/+^, RK *Ripk3*^−/−^, MW *Mlkl*^+/+^, MK Mlkl^−/−^

It was previously characterized that the necroptosis can initiate the activation of NLRP3 inflammasome in immune cells^5–7^. To determine the contributions of renal parenchymal cells and bone marrow (BM)-derived immune cells to the pathogenesis of IRI to CKD in vivo, we performed BM transplantation studies. *Ripk3*^+/+^ to *Ripk3*^+/+^ chimeric mice developed renal failure and serious tubular injury as determined by PAS staining at 2 and 14 days after IRI, but those characteristic symptoms were significantly reduced in *Ripk3*^+/+^ to *Ripk3*^*−/−*^ and *Ripk3*^*−/−*^ to *Ripk3*^*−/−*^ chimeras (Fig. 5a–c). However, *Ripk3*^*−/−*^ to *Ripk3*^+/+^ chimeric mice did not reveal any significant protection at days 2, but displayed protection at 14 days after IRI (Fig. 5a–c). The level of MCP-1 mRNA were upregulated in *Ripk3*^+/+^ recipients but not in *Ripk3*^*−/−*^ recipients regardless of the immune cell source at 2 days after IRI. Whereas, MCP-1 mRNA expression decreased in *Ripk3*^*−/−*^ to *Ripk3*^+/+^ mice at 14 days, compared with *Ripk3*^+/+^ to *Ripk3*^+/+^ chimerics (Fig. 5e). Similar results were performed in *Mlkl* background mice (Fig. 5f–h, j). These results indicated that immune cells did not significantly contribute to the early kidney injury after IRI, but after initiation of renal tubular necroptosis, more and more immune cells infiltrated the injury area and accelerated the progression of IRI to CKD.

At 2 months after IRI, *Ripk3*^+/+^ to *Ripk3*^+/+^ chimeric mice developed significant renal fibrosis, infiltrating leukocytes (Fig. 5a–d) and high levels of processed IL-1β and caspase1 activation (Fig. 5k). *Ripk3*^−/−^ to *Ripk3*^+/+^chimeric mice reduced renal fibrosis and inflammasome activation (Fig. 5a, d, k). Thus, BM-derived cells with *Ripk3* deficiency alleviated the inflammasome activation and renal fibrosis during the development of IRI to CKD. In contrast, the mice with *Ripk3*^−/−^ background, showed significantly reduced levels of renal fibrosis and inflammasome activation than the mice with *WT* background, irrespective of whether they were transplanted with *Ripk3*^+/+^or *Ripk3*^*−/−*^ bone marrow. However, the level of renal protection was strongest in *Ripk3*^−/−^ to *Ripk3*^−/−^ chimeras (Fig. 5a, d, k). Results were similar in experiments that were performed in *Mlkl*^+/+^ and *Mlkl*^−/−^ mice (Fig. 5f, i, l). These results might suggest that both parenchymal cells and immune cells contributed to the long-term renal outcome after IRI.

Taken together, these results indicated that renal parenchymal cell necroptosis is probably responsible for the renal injury in an early stage of AKI and then triggered inflammation, which subsequently accelerated necroptosis and trigger more inflammation in an auto-amplification loop, finally leading to renal fibrosis.

### RIPK3-induced PTC necroptosis after OGFD treatment is mediated by MLKL

**Fig. 6:**
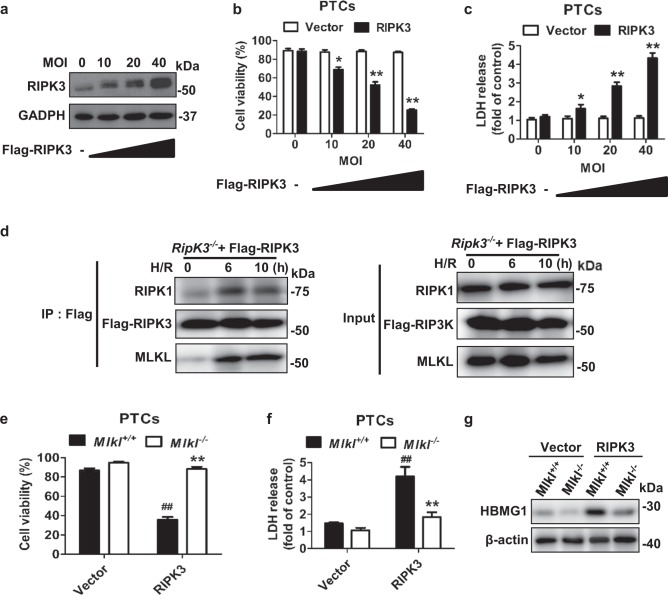
RIPK3, and MLKL is essential for OGFD-induced cell necroptosis in PTCs. **a**–**c** PTCs from mice were infected with different doses as the indicated MOI of Flag-RIPK3–expressing lentivirus for 40 h and then treated with OGFD for 8 h. **a** RIPK3 expression are shown by western blot. **b** The cell viability was assessed by PI staining. PI-positive cells indicated cell death. **c** LDH concentration in the medium were detected. **d** PTCs from *Ripk3*^+/+^ mice were infected with Flag-RIPK3–expressing lentivirus (MOI: 20) before stimulation with OGFD treatment for the indicated time. Cell lysates were immunoprecipitated with anti-Flag antibody (IP: Flag) and analyzed by immunoblotting with anti-RIPK1, anti-RIPK3, or anti-MLKL antibodies. Input, 5% of extract before immunoprecipitation (control). **e**–**g** *Mlkl*^+/+^ and *Mlkl*^−/−^ PTCs infected with RIPK3 lentivirus (MOI: 40) and treated with OGFD for 8 h. Cellular viability (**e**) and LDH (**f**) were detected. **g** HBMG1 in cell medium was detected by western blot. *n* = 3

The upregulated expression of RIPK3 could enhance necroptosis and participate in the pathogenesis of some disease models^8,16,31^. We further investigated the capacity of the elevated expression of RIPK3 in PTCs to enhance the susceptibility to OGFD-induced necroptosis. We found that RIPK3-overexpression in cultured PTCs by transfection (Fig. 6a) could lead to severe necroptosis in a dose-dependent manner, as shown by the cell viability and the increased LDH levels (Fig. 6b, c).

We further examined the involvement of some known necroptotic signal transduction events in OGFD-induced necroptosis. We reconstituted RIPK3 expression with in Flag-RIPK3 in *Ripk3*^*−/−*^ cells, and then used an anti-Flag antibody to perform immunoprecipitation of RIPK3. As shown in Fig. 6d, RIPK1 and MLKL were coimmunoprecipitated with RIPK3 in PTCs after OGFD treatment, demonstrating that the formation of necrosomes in OGFD-treated PTCs. Moreover, it was noticed that *Mlkl* deficiency attenuated OGFD-induced necroptosis (Fig. 6e, f) and reduced OGFD-induced release of HMGB1 into the cell culture media in RIPK3-overexpression PTCs (Fig. 6g). Thus, RIPK3 is a key factor in mediating necroptosis induced by OGFD *via* its interaction with MLKL.

### RIPK3-MLKL-dependent NLRP3 inflammasome activation in macrophages could be driven by renal tubular cell necroptosis

**Fig. 7:**
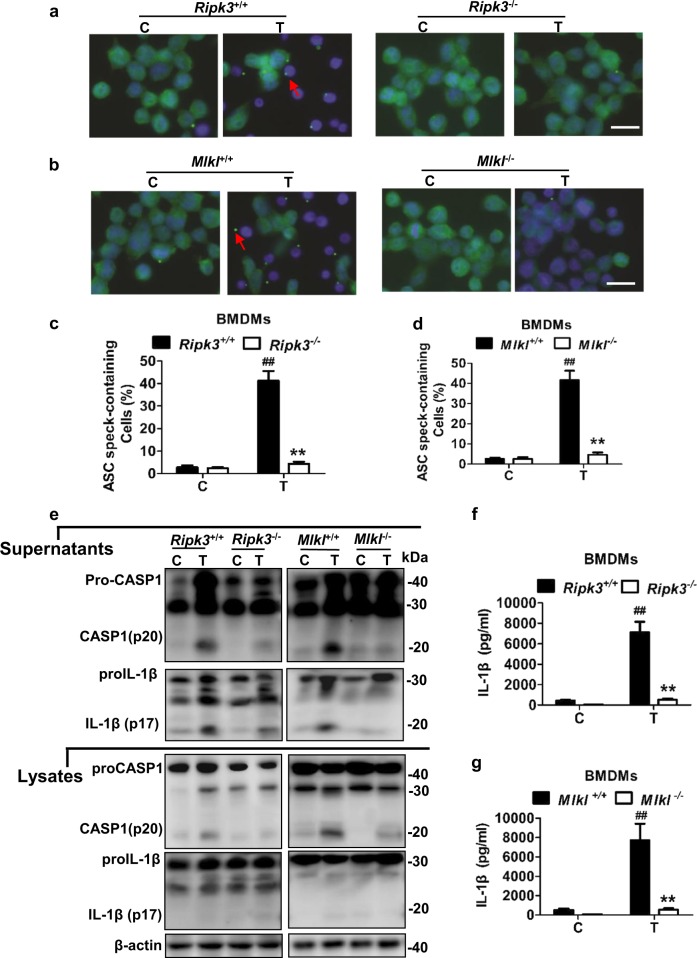
RIPK3 and MLKL activates the NLRP3 inflammasome in macrophages driven by tubular cell necroptosis. *Ripk3*^+/+^, *Ripk3*^*−/−*^, *Mlkl*^+/+^ and *Mlkl*^*−/−*^ BMDMs were pre-incubated with LPS (100 ng/ml) for 4 h and then were treated with supernatants from necrotic PTCs, containing 100 nM Cp.A. **a**, **b** ASC speck formation (Alexa488, green), and nuclei (DAPI, blue) were assessed by fluorescence microscopy. Bar = 50 μM. **c**, **d** Specks as red arrow showed were automated and analyzed by Image J and represented as a percentage of total specks measured. *n* = 5. **e** Supernatants and total cell lysates were analyzed by western blot as indicated. *n* = 4. C: control, T: treatment. **f**, **g** IL-1β levels in supernatants were assessed for by ELISA

Necroptotic cell lysis and resultant release of proinflammatory mediators are thought to cause inflammation in necroptotic disease models^6,8,16,31,32^. We hypothesized that necroptotic PTC lysis could induce NLRP3 signaling inflammasome activation in macrophages. To mimic the in vivo environment after IRI, we used oxygen-glucose and fetal bovine serum deprivation (OGFD) to induce necroptosis in RIPK3-overexpressing PTCs. We used OGFD-induced necroptosis in RIPK3-overexpressing PTCs. Then we collected the supernatant after OGFD treatment to stimulate bone marrow-derived macrophages (BMDMs). The formation of NLRP3-induced ASC specks was visualized in WT BMDMs, but significantly prevented in *Ripk3* or *Mlkl* deficiency BMDMs (Fig. 7a–d). The processing of caspase1 (Fig. 7e) and large amounts of IL-1β was detected in BMDMs after this stimuli, which could be attenuated by *Ripk3* or *Mlkl* deficiency (Fig. 7e–g). This suggest that inflammasome activation in inflammatory cells could be triggered and enhanced by tubular cell necroptosis.

### RIPK3-enhanced PTC inflammasome activation after OGFD treatment is mediated by MLKL

**Fig. 8:**
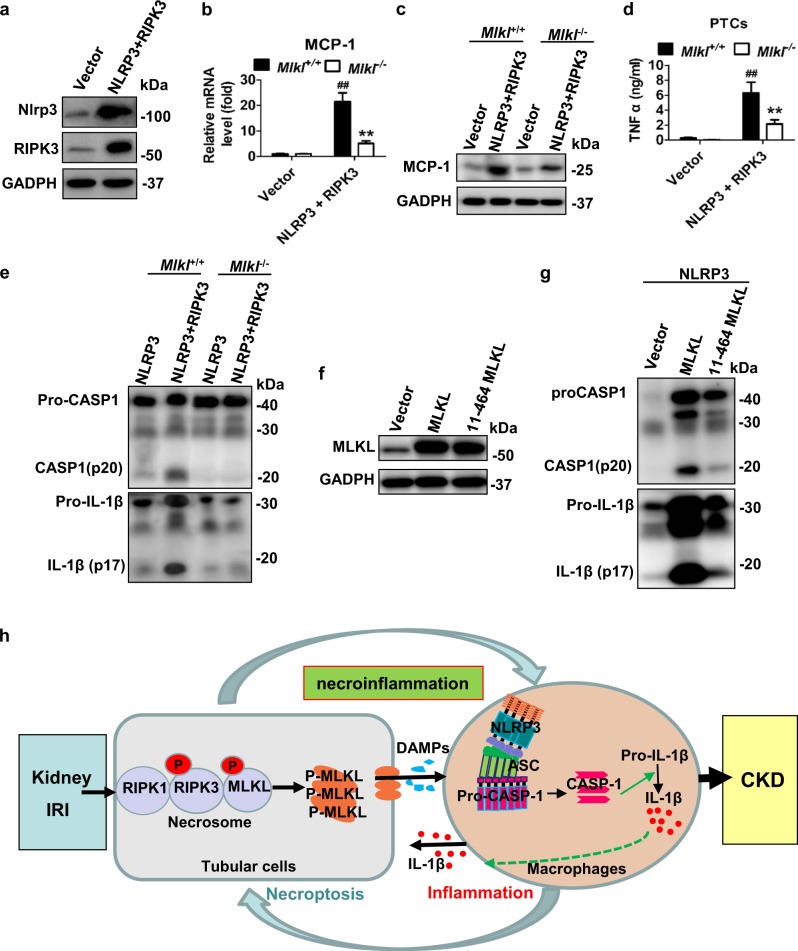
RIPK3-enhanced necroptosis and promoted caspase-1 activation and IL-1β secretion. **a**–**e** PTCs were infected with lentivirus encoding vector or NLRP3 (MOI: 30) or NLRP3 (MOI: 30) and RIPK3 (MOI: 30) for 40 h, and then treated with OGFD for 8 h. **a** The levels of NLRP3 and RIPK3 are determined by western blot. **b** MCP-1 mRNA and (**c**) MCP-1 protein expression in PTCs were analyzed. **d** TNFα levels in supernatants were detected by ELISA. **e** IL-1β and caspase1 in cell supernatants were analyzed by western blot as indicated. **f**, **g** PTCs were infected with lentivirus encoding NLRP3 (MOI:30) and MLKL (MOI: 30) or 11-464MLKL (MOI: 30). MLKL expression, IL-1β and caspase1 in cell supernatants were analyzed by western blot. *n* = 3. **h** Proposed model of necroptosis and necroptosis-driven inflammasome activation in the progression of IRI to CKD

Since renal NLRP3 also increased in vivo after IRI, we determined whether RIPK3 could promote inflammasome activation and IL-1β secretion in NLRP3- overexpressing PTCs. Strikingly, MCP-1 expression and TNFα secretion was significantly upregulated after double overexpression of NLRP3 and RIPK3 in PTCs, which could be reduced by *Mlkl* deficiency (Fig. 8a–d). RIPK3 overexpression enhanced the sensitivity of PTCs to H/R-induced inflammasome activation in cultured NLRP3-overexpressing PTCs. The level of processed caspase-1 and IL-1β significantly increased in double NLRP3 and RIPK3-overexpressing PTCs after OGFD, which was attenuated by *Mlkl* deficiency (Fig. 8e). N-terminal domain is necessary for MLKL oligomerization and redistribution from the cytoplasm into cell membrane, which is correlated with the secretion of IL-1β^24^. We determined if the MLKL translocation to cell membrane is also necessary for PTC IL-1β secretion. MLKL could enhance IL-1β secretion in PTCs with NLRP3-overexpression, but 10-amino-acid deletion from the N-terminus of MLKL could not (Fig. 8f, g). Taken together, these data indicated that the increase of RIPK3, MLKL and NLRP3 expression could promote MCP-1 expression to attract inflammatory cell infiltration and PTC itself NLRP3 inflammasome activation and inflammatory cytokines secretion post IRI. We have demonstrated before that inflammatory cytokine upregulation could promote necroptosis progression^16^, suggesting a positive feedback loop involving necroptosis and inflammation contributed to IRI progression.

## Discussion

The mechanisms underlying the transition from AKI to CKD is still unclear. Increasing evidences have shown that necroptosis rather than apoptosis, has a predominant role at the early stage of pathological progression of various types of AKI^12,13,15,16^. Necroptosis is a highly orchestrated process, depending on the crucial initiating factors, RIPK1, RIPK3 and MLKL^9,33,34^. Blocking necroptotic pathways by pharmacological inhibition or deletion of *Ripk3* or *Mlkl* alleviates acute renal injuries at an early stage induced by cisplatin and IR^12–16^. It has also been demonstrated that non-necroptotic role of RIPK3 and MLKL contributes to AKI progression and CKD^15,35^. Here we have showed that renal protection after IRI was comparable in *Ripk3* deficiency, *Mlkl* deficiency and *Ripk3/Mlkl* double deficiency mice, suggesting that MLKL acts an important downstream effector of RIPK3 in kidney injury induced by IRI. These data support the conclusion that RIPK3-dependent; MLKL-independent effects are of minor importance in the progression renal IRI^15^. In addition, we further demonstrated that inhibiting RIPK3/MLKL-dependent necroptosis could suppress the progression of AKI to CKD.

The NLRP3 inflammasome is known to be activated by a variety of non-microbial danger signals released by the necrotic cells^6,20^, and is considered as an important pathway that sustains the inflammation–fibrosis cycle in CKD^3,4^. Here we found that deletion of RIPK3 or MLKL alleviated the NLRP3 inflammasome activation and IL-1β release in kidney after IRI corresponding with the reduction of renal fibrosis. Furthermore, we showed that *Ripk3*^−/−^ to *Ripk3*^+/+^ or *Mlkl*^−/−^ to *Mlkl*^+/+^ chimeric mice developed similar renal failure and serious tubular injury at an early stage of IRI compared with chimeric mice from WT donors. In contrast, chimeras of *Ripk3* or *Mlkl* knockout recipients were resistant to IRI at early stage regardless of the transplanted bone marrow cells. Expression of MCP-1 was increased in WT-WT and KO-WT mice but not in WT-KO or KO-KO mice at day 2 post IRI, suggesting that tubular injury is responsible for the expression of MCP-1 capable of attracting infiltrating inflammatory cells at an early stage after IRI. Therefore, RIPK3/MLKL-dependent necroptosis in renal tubular cells is the predominant factor in initiation of IRI and then secondarily triggers the inflammation, which could accelerate necroptosis in a positive feedback loop and promoted the progression of IRI to CKD. However, in late stage, 14 days after IRI, *Ripk3*^−/−^ to *Ripk3*^+/+^ or *Mlkl*^−/−^ to *Mlkl*^+/+^ chimeric mice showed less renal tubular injury and less inflammation. Moreover, *Ripk3*^−/−^ to *Ripk3*^*−/−*^ chimeric mice displayed better protective effect in the long term after IRI than *Ripk3*^+/+^ to *Ripk3*^−/−^ by more strongly inhibiting inflammasome activation and IL-1β release, suggesting that deletion of *Ripk3* or *Mlkl* in immune cells contributed to alleviating AKI to CKD by preventing NLRP3 inflammasome activation. These data indicated that the progression of IRI to CKD is prevented in *Ripk3* or *Mlkl* deficiency mice because of inhibiting the initiation of renal tubular necroptosis at an early stage and preventing necroinflammation at the later stage.

It has been shown that RIPK3-MLKL necroptotic signaling activates the NLRP3 inflammasome when caspase 8 was inhibited^17,18^. In vivo, caspase 8 activation was actually inhibited after IRI. Interestingly, *Ripk3* or *Mlkl* deficiency mice (43-min ischemia) displayed reduced NLRP3 inflammasome activation after the similar initial renal injury with WT mice (35-min ischemia), suggesting that both RIPK3 and MLKL contributed to inflammasome activation during the progression of IRI. We also found that supernatants from necrotic PTCs with RIPK3-overexpression induced by H/R actually enhanced caspase1 activation and IL-1β secretion, but did not trigger cell death in bone marrow-derived macrophages. Recently it has been identified that RIPK3/MLKL-mediated NLRP3 inflammasome activation and IL-1β secretion by necroptotic stimuli occurs in a cell-intrinsic manner before macrophages lysis. This event is separable from pyroptosis^24^. We also showed that *Ripk3* or *Mlkl* deficiency remarkably abrogated the ability of necroptotic cell supernatants-induced caspase1 activation and IL-1β secretion in macrophages. Thus, the activation of immune cell inflammasome could be driven by necroptotic tubular cells under IRI condition.

Our studies also demonstrated that increased renal tubular RIPK3 and MLKL expression in IRI contributed to initiation of necroptosis and enhanced NLRP3 inflammasome activation. The protein level of RIPK3, MLKL and NLRP3 were upregulated in renal proximal tubules after IRI, although how their expressions increased was still unknown. Overexpression of RIPK3 and MLKL in PTCs in vitro actually enhanced PTC necroptosis. Interestingly, in vivo, NLRP3 upregulation and the inflammasome activation in renal proximal tubules could also be detected after IRI. In vitro, RIPK3 actually promoted caspase1 activation and IL-1β maturation after OGFD treatment in NLRP3-overexpression PTCs, which could be attenuated by MLKL. Thus, renal tubular cells themselves performed the function of inflammasome activation and secretion of inflammatory factors when NLRP3 increased, which could be enhanced by RIPK3 and MLKL. We have demonstrated before that inflammatory cytokine upregulation could accelerate necroptosis progression, suggesting a positive feedback loop involving RIPK3-MLKL-dependent necroptosis and inflammation contributed to IRI progression. (Fig. 8h). However, necroptosis induced the secretion of numerous proinflammatory cytokines that could in turn induce several other forms of regulated necrosis. Therefore, deletion of *Ripk3* or *Mlkl* did not completely block the development of IRI to CKD.

In summary, our study delineates the cell death related mechanisms underlying the progression of AKI to CKD. Our findings showed that RIPK3-MLKL-dependent necroptosis triggering NLRP3 inflammasome activation in an auto-amplification loop, resulting in necroinflammation contributes to AKI progression to CKD. This indicates that blockade of RIPK3/MLKL signaling could be a promising strategy for clinical therapy of AKI to CKD.

## Materials and methods

### Animal models and experiments

*Ripk3*^−/−^ and *Mlkl*^−/−^ mice under C57BL/6 background were gifts from Jiahuai Han’s lab, School of Life Sciences, Xiamen University, China. Mice were housed in a specific pathogen-free facility. All experiments were approved by Laboratory Animal Management and Ethics Committee of Fujian Medical University, in accordance with the Chinese Guidelines on the Care and Use of Laboratory Animals. *Ripk3* and *Mlkl* double deficiency (*Ripk3*^−/−^*Mlkl*^−/−^) mice were generated from *Ripk3*^+/−^ and *Mlkl*^+/−^ under C57BL/6 background mice. Mice underwent the described procedure^9^ with 35-min, 40-min or 43-min bilateral renal pedicle clamping before reperfusion and were observed for 12 weeks.

For chimera studies, bone marrow (BM) cells were isolated from donor mouse femurs and tibias. Recipient male mice were irradiated by lethal X-ray irradiation (7.5 Gy), 2 × 10^6^ donor BM cells were injected into recipients *via* the tail vein. 2, 14 days, 2 months after IRI, mice were killed and the kidneys were removed. Kidneys were dissected for flash-frozen, paraffin embedding, and electron microscopy studies.

### Reagents and antibodies

Anti-RIPK1, anti-RIPK3, and anti-MLKL antibodies used in the present study were kindly provided by Prof. Jianhuai Han. Anti–mouse IL-1β antibody (5129–100; BioVision), anti-mouse caspase-1 p20 (AG-20B-0042-C100; AdipoGen), anti-ASC antibody (sc-22514; Santa Cruz Biotechnology), anti-NLRP3 antibody (ab214185; abcam); anti-GAPDH (3781; ProSci), anti-α-SMA (A5228; Sigma-Aldrich), anti-p-Smad3 (ab52903; Abcam), anti-collagen I (ab34710; abcam), and anti-tubulin (T9026; Sigma-Aldrich), anti-MCP-1(ab25124; abcam), anti-ICAM (ab25375; abcam) were used for western blotting. Anti-F4/80 antibody [CI: A3-1] (ab6640, abcam) was used for immunohistochemistry. Mouse TNFα ELISA Kit ab100747 was used for detecting kidney TNFα secretion.

### Immunohistochemistry

Tissue sections were fixed by paraffin-embedding. Routine protocols were used for immunohistochemical stainings. Briefly, sections were incubated with the F4/80 antibody at 1:50 dilution for 1 h at room temperature, followed by goat anti-rat IgG H&L (HRP) secondary antibody (ab97057, abcam). For morphologic quantifications, 10 random visual fields were analyzed per kidney section. The number of F4/80-positive cells were determined with Image J software.

### Immunofluorescence

After treatments, macrophages were plated on coverslips and then fixed in 4% paraformaldehyde for 15 min. Cells were permeabilized by 0.25% Triton X-100 in PBS for 10 min. After blocking with 10% normal goat serum, cells were incubated with anti-ASC antibody, followed by secondary antibodies. Finally, cells were stained with DAPI and mounted. Images were acquired using Fluorescence Microscope (Leica).

### Cell culture

Proximal tubules were freshly isolated as described in our previous studies. After approximately 4–5 days, the cultured primary proximal tubular cells (PTCs) were used in experiments at this point. HEK293T cells (gift from Pro. Jiahuai Han) were grown in DMEM containing 10% glucose, 10% fetal bovine serum, penicillin and streptomycin. BMDMs were obtained from the tibia and femur and differentiating bone marrow progenitors in RPMI 1640 containing 30% L929-conditional medium for 7 days.

To stimulate PTC necroptosis mimicing the in vivo condition after IRI, PTCs from mice were treated with oxygen-glucose-BSA deprivation (OGBD) for different hours as indicated and finally replaced with normal culture condition.

To detect if necrotic PTCs could trigger NLRP3 inflammasome in BMDMs, PTCs from mice were infected with vector or RIPK3–expressing lentivirus and then treated with OGBD for 10 h. The supernatants were collected and added with glucose and BSA, and then labeled as supernatant A, which were used to induce NLRP3 inflammasome activation in BMDMs. To activate the NLRP3 inflammasome in BMDMs, BMDMs were primed with 1 μg/ml LPS for 4 h and then treated with Cp.A (100 nM) in the supernatant A as described, followed for 8 h.

### Lentivirus preparation and infection

For the packaging of lentivirus, 293 T cells were co-transfected with expressing plasmid and lentivirus-mix plasmids (PMDL/REV/VSVG) by calcium phosphate precipitation. pBOBI-RIPK3, pBOBI-Flag-RIPK3, pBOBI-MLKL, pBOBI-11-464-MLKL and pBOBI-NLRP3, were provided by Prof. Jianhuai Han. The virus-containing medium was harvested 40–48 h later and added to cells in presence of 10 mg/ml polybrene. Infectious medium was changed 12–14 h later, and the infected cells were further used for experiments after 36–48 h.

### Immunoprecipitation

*Ripk3*^−/−^ PTCs were transfected with an expression vector encoding Flag-RIPK3 for 36 h prior to treatment with OGBD as the indicated time. After treatment, Lysis buffer was added. Immunoprecipitations were performed by using anti-Flag beads as described^16^. Western blotting of immunoprecipitates and cell lysates was performed by using anti-RIPK3, anti-RIPK1, and anti-MLKL as indicated.

### Western blotting

The freshly isolated renal proximal tubules or primary cultured PTCs were harvested and then lysed with 1.2x SDS buffer immediately. Total tissue protein lysates were placed in lysis buffer complete with protease inhibitors, and then homogenized. Protein concentrations were determined using the Bradford protein assay and then 5× SDS buffer was added. The lysates were separated by SDS-PAGE and transferred to polyvinylidene fluoride membranes (EMD Millipore) for western blotting analysis with the appropriate antibodies.

### RNA analysis

Total RNA was obtained from PTCs by RNA-iso reagent (TakaRa). Total RNA was reverse-transcribed to cDNA using Reverse Transcription Kit (BGI, Shenzheng, China). The levels of ICAM-1, MCP-1 and β-actin were determined by SYBRGreen I Real-time quantitative PCR in a CFX96 real-time RT-PCR detection system (Bio-Rad). PCR amplification was carried out for 42 cycles. The following primer sequences were used: MCP-1 (forward: 5′-GAGGACAGATGTGGTGGGTTT-3′, reverse: 5′-AGGAGTCAACAGCTTTCTCTT-3′); ICAM-1 (forward: 5′-GTGATGCTCAGGTATCCATCCA-3′, reverse: 5′- CACAGTTCTCAAAGCACAGCG-3′); GAPDH (forward: 5′-TGTGTCCGTCGTGGATCTGA-3′, reverse: 5′-CCTGCTTCACCACCTTCTTGA-3′). TNFα (forward: 5′-CCTCTCTCTAATCAGCCCTCTG-3′, reverse: 5′-GAGGACCTGGGAGTAGATGAG-3′).

### Statistical analysis

All experiments were independently performed in triplicate as a minimum. All the data are expressed as means ± SEM. Unpaired *t*-test or one-way ANOVA was used to statistical analyses. *P* *<* 0.05 was considered to indicate statistical significance.

## Electronic supplementary material

Both RIPK3 and MLKL contributed to renal fibrosis after IRI

Ripk3-/- and Mlkl-/- mice reduced macrophage recruitment in tubulointerstitium post IRI

Ripk3 or Mlkl deficiency reduced NLRP3 inflammasome activation post IRI
