# Supplementary material for: RIPK3-MLKL-mediated necroinflammation contributes to AKI progression to CKD
Source: Cell Death Dis. 2018 Aug 29;9(9):878. doi: 10.1038/s41419-018-0936-8 (PMC6115414; doi:10.1038/s41419-018-0936-8)
Supplement: Supplementary file 1 — Both RIPK3 and MLKL contributed to renal fibrosis after IRI [file 41419_2018_936_MOESM1_ESM.pdf]

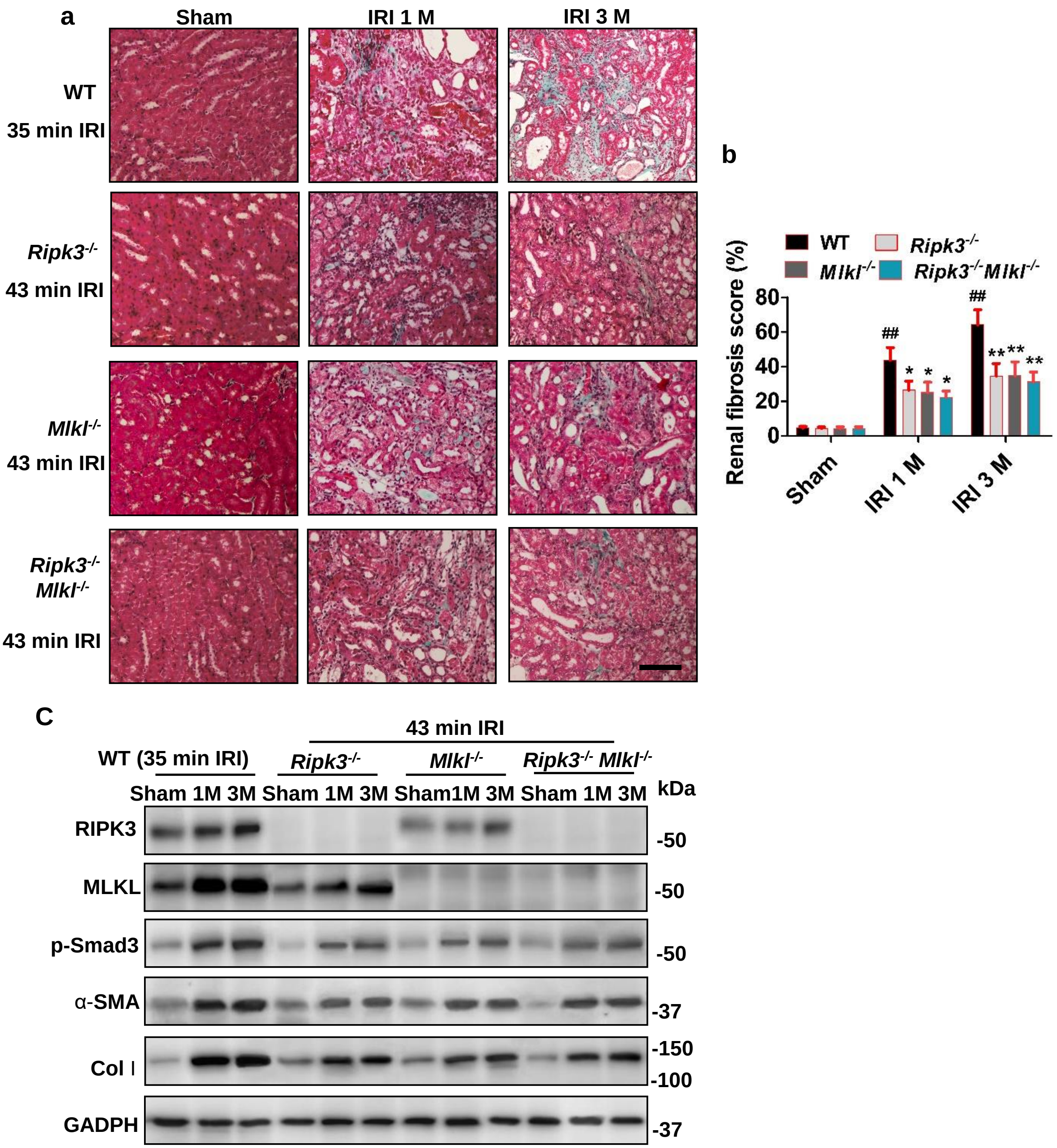

Fig. S1 Both RIPK3 and MLKL contributed to renal fibrosis after IRI. WT mice were subjected to renal IRI with 35-min ischemia and all knock out mice were subjected to IRI with 43-min ischemia. n=6. (a) Kidney morphology by Masson's trichrome staining. Representative micrographs in kidney sections. Bar=100 μM. (b) Renal fibrosis scores obtained from Masson's trichrome-stained sections were analyzed with Image J. n=6. $^{\#\#}$*P*<0.01 *versus* WT sham group; **P*<0.05 *versus* WT IRI,***P*<0.01 *versus* WT IRI. (c) Representative immunoblot for RIPK3, MLKL, p-Smad3, α-SMA, Col Ⅰ, and β-actin by Western blot in total kidney lysates.
