# Supplementary material for: RIPK3-MLKL-mediated necroinflammation contributes to AKI progression to CKD
Source: Cell Death Dis. 2018 Aug 29;9(9):878. doi: 10.1038/s41419-018-0936-8 (PMC6115414; doi:10.1038/s41419-018-0936-8)
Supplement: Supplementary file 2 — Ripk3-/- and Mlkl-/- mice reduced macrophage recruitment in tubulointerstitium post IRI [file 41419_2018_936_MOESM2_ESM.pdf]

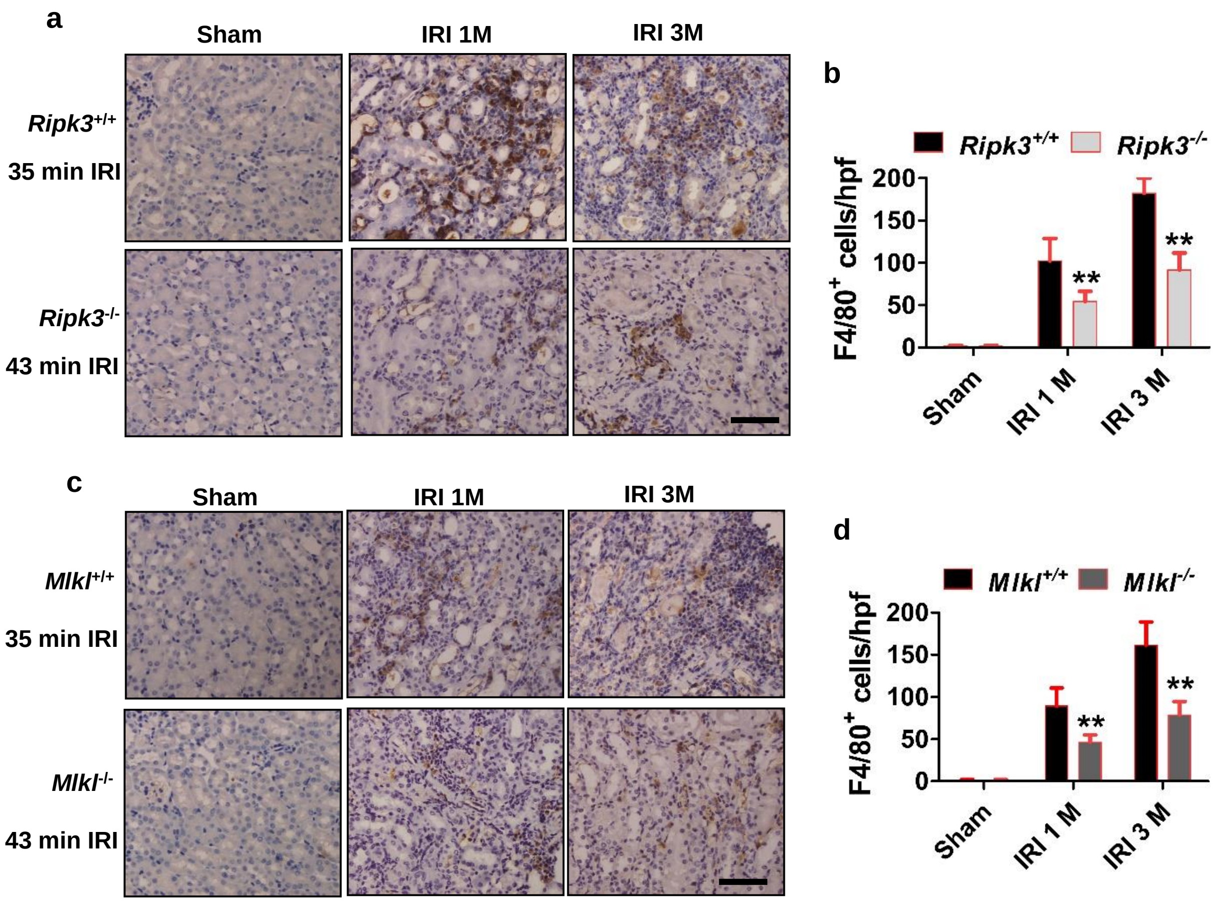

Fig. S2 *Ripk3*$^{-/-}$ and *Mlkl*$^{-/-}$ mice reduced macrophage recruitment in tubulointerstitium post IRI. WT mice underwent renal IRI with 35-min ischemia and all gene knockout mice underwent renal IRI with 43-min ischemia. n=6. (a, c) Representative images of immunohistochemistry of kidney tissues with the monocytes-macrophage marker F4/80^{+} at 1, 3 months following IRI. Bar=100 μM. (b, d) The number of F4/80-positive cells per hpf was quantified. ***P*<0.01 *versus* WT group.
