# Supplementary material for: RIPK3-MLKL-mediated necroinflammation contributes to AKI progression to CKD
Source: Cell Death Dis. 2018 Aug 29;9(9):878. doi: 10.1038/s41419-018-0936-8 (PMC6115414; doi:10.1038/s41419-018-0936-8)
Supplement: Supplementary file 3 — Ripk3 or Mlkl deficiency reduced NLRP3 inflammasome activation post IRI [file 41419_2018_936_MOESM3_ESM.pdf]

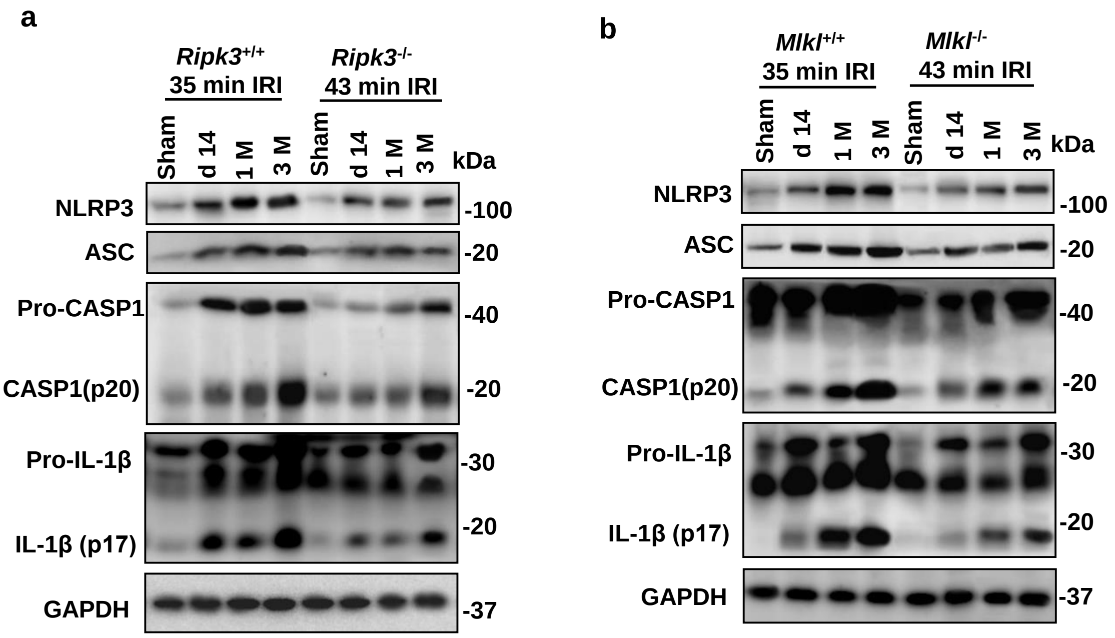

Fig. S3 ***Ripk3* or *Mlkl* deficiency reduced NLRP3 inflammasome activation post IRI.** All mice underwent renal IRI as Fig. S2. (a, b) Expression of NLRP3, ASC, the active caspase-1 and the mature (processed) IL-1β in total lysates of kidneys were assessed by Western blot analysis. GADPH was used as the loading control. n=4.
